# A Low Dose of Pure Cannabidiol Is Sufficient to Stimulate the Cytotoxic Function of CIK Cells without Exerting the Downstream Mediators in Pancreatic Cancer Cells

**DOI:** 10.3390/ijms23073783

**Published:** 2022-03-29

**Authors:** Francesca Garofano, Amit Sharma, Hinrich Abken, Maria A. Gonzalez-Carmona, Ingo G. H. Schmidt-Wolf

**Affiliations:** 1 Department of Integrated Oncology, Center for Integrated Oncology (CIO), University Hospital Bonn, 53127 Bonn, Germany; francesca.garofano@ukbonn.de (F.G.); amit.sharma@ukbonn.de (A.S.); 2 Department of Neurosurgery, University Hospital Bonn, 53127 Bonn, Germany; 3 RCI Regensburg Center for Interventional Immunology, Department Genetic Immunotherapy, University Hospital Regensburg, 93053 Regensburg, Germany; hinrich.abken@ukr.de; 4 Department of Internal Medicine I, University Hospital Bonn, 53127 Bonn, Germany; maria.gonzalez-carmona@ukbonn.de

**Keywords:** cytokine-induced killer cells, cannabidiol, pancreatic cancer

## Abstract

Despite numerous studies conducted over the past decade, the exact role of the cannabinoid system in cancer development remains unclear. Though research has focused on two cannabinoid receptors (CB1, CB2) activated by most cannabinoids, CB2 holds greater attention due to its expression in cells of the immune system. In particular, cytokine-induced killer cells (CIKs), which are pivotal cytotoxic immunological effector cells, express a high-level of CB2 receptors. Herein, we sought to investigate whether inducing CIK cells with cannabidiol can enhance their cytotoxicity and if there are any possible counter effects in its downstream cascade of phosphorylated p38 and CREB using a pancreatic ductal adenocarcinoma cell line (PANC-1). Our results showed that IL-2 modulates primarily the expression of the CB2 receptor on CIK cells used during ex vivo CIK expansion. The autophagosomal-associated scaffold protein p62 was found to co-localize with CB2 receptors in CIK cells and the PANC-1 cell line. CIK cells showed a low level of intracellular phospho-p38 and, when stimulated with cannabidiol (CBD), a donor specific variability in phospho-CREB. CBD significantly decreases the viability of PANC-1 cells presumably by increasing the cytotoxicity of CIK cells. Taken together, in our preclinical in vitro study, we propose that a low effective dose of CBD is sufficient to stimulate the cytotoxic function of CIK without exerting any associated mediator. Thus, the combinatorial approach of non-psychoactive CBD and CIK cells appears to be safe and can be considered for a clinical perspective in pancreatic cancer.

## 1. Introduction

Cancer is a heterogeneous disease where multiple overlapping molecular pathways are involved [1]. In fact, the combinatorial role of various inhibitors/compounds is constantly being explored along with several therapeutic approaches in order to overcome the impact of this disease. In this scenario, the pivotal role of cytokine-Induced killer (CIK) cells as a cellular antitumor therapy cannot be ignored. CIK cells share phenotypic and functional properties of both T cells and NK cells, and are easily expandable to test in culture. Moreover, CIK therapy has been proven effective and safe in the treatment of various cancers and recently celebrated 30 years of successful implementation [2]. To mention, CIK cells display encouraging synergistic effects when combined with cancer associated inhibitors/blockades [3,4]. Since cannabinoid receptors have been the subject of intensive cancer research [5,6], in particular, cannabinoid receptor 2 (CB2) holds greater attention due to its expression in cells of the immune system where CIK cells also play an important role. To mention, recently, we have also shown that CIK cells express high levels of CB2 receptor compared to PBMCs [7]. 

Specifically, in pancreatic cancer (PC), where several clinical trials based on CIK cell therapy have yielded encouraging results [8,9], cannabinoids have also been reported to inhibit pancreatic cancer cell growth in vitro and in vivo through various mechanisms [10]. PC as a fatal illness is usually recognized late in the metastatic stage, primarily due to the anatomic localization of the pancreas and the nonspecific nature of the symptoms. Despite all clinical and molecular advances [11,12,13], PC still harbors a very poor prognosis and high mortality rate [14]; therefore, it is of prime interest to set up new strategies. Previously, synthetic cannabinoid derivatives have been shown to induce cell death in pancreatic MIA PaCa-2 cells via a receptor-independent mechanism [15]. Moreover, human PC cell lines and tumor biopsies have been shown to express higher levels of cannabinoid receptors compared to normal pancreatic tissue [16]. The same study also demonstrated that cannabinoids lead to apoptosis of pancreatic tumor cells via a CB2 receptor and de novo synthesized ceramide-dependent up-regulation of p8 and the endoplasmic reticulum stress–related genes ATF-4 and TRB3. Interestingly, the scaffold/phagosomal protein p62/SQSTM1 has recently been identified as part of the CB2 receptor interactome in transfected HEK293 cells [17]. Likewise, an independent interesting study demonstrated that CB2 induces the phosphorylation of p38 MAPKs, downstream CREB phosphorylation and induction of IL-6, IL-10 cytokine secretion in human primary leukocytes [18]. It is noteworthy that the exact mode of action of these cannabinoid receptors is unclear, despite being involved in immune system like CIK cells, their possible crosstalk and underlying mechanisms remain unexplored.

Considering this, herein, we sought to investigate whether inducing CIK cells with cannabidiol can enhance their cytotoxicity. While we focused primarily on PC in this study, we also used myeloma cells as a proof of concept. Using multiple methods (Flow cytometry, immunohistochemistry, laser cell microscopy, cytotoxicity based in vitro assays), we address the cannabidiol modulation along with CIK cells.

## 2. Materials and Methods

### 2.1. Generation of Cytokine-Induced Killer Cells

Peripheral blood mononuclear cells (PBMCs) were isolated from blood samples of healthy donors after obtaining the approval of the ethics committee of the University Hospital Bonn, including a signed informed consent from the volunteers. The isolation was carried out on the same day or kept overnight at 4 °C for further use on the next day. Briefly, the blood was mixed with Dulbecco’s phosphate-buffered saline (DPBS; PAN BIOTECH, Aidenbach, Germany)-ethylenediaminetetracetic acid (EDTA; Life Technologies, PAA, Cölbe, Germany) (1:250) in a 50 mL falcon tube at a ratio of 1:1 and then transferred to another falcon tube containing Lymphoprep density gradient medium (Pancoll) (PAN BIOTECH, Aidenbach, Germany) in order to perform a gradient density centrifugation. The collected PBMC were washed twice with DPBS-EDTA. Erythrocytes were lysed and washed away with red blood cell (RBC) lysis buffer (Biolegend, San Diego, CA, USA) and subsequently washed with DPBS-EDTA. The cells obtained were then seeded at a density of 1–2 × 10^6^ cells/mL in a T-175 flask containing 40 mL of culture medium RPMI 1640 (PAN BIOTECH, Aidenbach, Germany) supplemented with 10% newborn calf serum (NCS) (Sigma, St. Louis, MO, USA), 1% penicillin and streptomycin P/S (Gibco, Gaithersburg, MD, USA), and 1 M Hepes (PAN BIOTECH, Aidenbach, Germany). The generation of CIK cells was primed by adding 20 μL of IFN-γ (ImmunoTools GmbH, Friesoythe) (2000 U/μL) on day 0. On the next day, 100 μL of IL-1 (ImmunoTools GmbH, Friesoythe, Germany) (40 U/μL), 2 μL of anti-CD3 antibody (eBioscience, Thermo Fisher Scientific, Inc., San Diego, CA, USA) (1 mg/mL), as well as 24 μL of IL-2 (ImmunoTools GmbH, Friesoythe, Germany) (1000 U/μL) or IL-15 (40 ng/mL) (ImmunoTools GmbH, Friesoythe, Germany) were added into the cells. Every third day, half of the medium was exchanged and 600 U/mL IL-2 or IL-15 (40 ng/mL) were added. The CIK cells were expanded for 14 days ex vivo and used for co-culturing experiments.

### 2.2. Cell Line and Cell Culture

In the current study, we utilized two cell lines, one sourced from pancreatic cancer (Pancreatic ductal adenocarcinoma cell line: PANC-1) and another from multiple myeloma (U-266), both obtained from Leibniz Institute DSMZ Deutsche Sammlung von Mikroorganismen und Zellkulturen. Both cell lines were cultured in RPMI 1640 (PAN BIOTECH, Aidenbach, Germany) supplemented with 10% newborn calf serum (NCS) (Sigma, St. Louis, MO, USA) and 1% penicillin and streptomycin P/S (Gibco, Gaithersburg, MD, USA) at 37 °C in a humidified atmosphere with 5% CO_2_.

### 2.3. Cytotoxicity Assay Based on CCK-8

After co-culture with CIK cells for 24 h, the cells’ viability of PANC-1, U-266 tumor cell lines and CIK cells exposed to different concentrations (1–20 μM, at 37 °C) of pure cannabidiol (CBD 100%) (Santa Cruz Biotechnologie, Heidelberg, Germany) was determined by CCK-8 based method. Briefly, the effector cells (CIK cells) were co-cultured with target cells (PANC-1, U-266) at the effector to target (E:T) ratios of 10:1 and seeded into flat bottom 96-well plates. Next, 10 μL of CCK-8 reagent (Dojindo, Kumamoto, Japan) were added in each well, according to the manufacturer’s instructions. After incubation for 1 h, the absorbance of each well was measured at 450 nm using a microplate reader. All the experiments were performed in triplicates. This particular experiment was replicated three times with CIK cells from three different donors.

### 2.4. LDH Assay

A commercial CyQUANT LDH Cytotoxicity Assay Kit (ThermoFisher, Waltham, MA, USA) was used, according to the manufacturer’s instructions. Here again, PANC-1, U-266 cell lines and CIK cells were exposed to different concentrations (1–20 μM, 24 h at 37 °C) of pure cannabidiol (CBD) (Santa Cruz Biotechnologie, Heidelberg, Germany). The effector cells (CIK cells) were co-cultured with target cells (PANC-1, U-266) at the effector to target (E:T) ratios of 10:1 and seeded into 48-well plates. At the end of incubation, 25 μL of each sample were transferred to a 96-well flat bottom plate and 25 μL of the reaction mixture were added. The absorption of the released LDH was then measured using a microplate reader at 490 nm and 680 nm. To determine LDH activity, the 680 nm absorbance value was subtracted from the 490 nm absorbance value. All experiments were performed in triplicates and replicated three times with CIK cells from three different donors. To calculate the % cytotoxicity, the following equation was applied to the corrected values:

$$\%\mathrm{cytotox}.=\frac{\mathrm{Experimental}\;\mathrm{value}\;-\;\mathrm{Effector}\;\mathrm{Cells}\;\mathrm{Spontaneous}\;\mathrm{Control}\;-\;\mathrm{Target}\;\mathrm{Cells}\;\mathrm{Spontaneous}\;\mathrm{Control}}{\mathrm{Target}\;\mathrm{Cells}\;\mathrm{Maximum}\;\mathrm{Control}\;-\;\mathrm{Target}\;\mathrm{Cells}\;\mathrm{Spontaneous}\;\mathrm{Control}\;}\times 100$$

To calculate the % cytotoxicity of CBD on tumor cells, the following equation was applied to the corrected values:

$$\%\mathrm{cytotox}.=\frac{\mathrm{Compound}-\mathrm{treated}\;\mathrm{LDH}\;\mathrm{activity}\;-\;\mathrm{Spontaneous}\;\mathrm{LDH}\;\mathrm{activty}}{\mathrm{Maximum}\;\mathrm{LDH}\;\mathrm{activty}\;-\;\mathrm{Spontaneous}\;\mathrm{LDH}\;\mathrm{activty}\;}\times 100$$

### 2.5. Immunocytochemistry

PANC-1 and CIK cells were plated on poly-L-Lysine (Sigma, St. Louis, MO, USA) coated glass cover slips with their respective cell culture medium. After 2 h incubation at 37 °C, cells were washed with DPBS (PAN BIOTECH, Aidenbach, Germany) and stained with the lectin WGA conjugated with Texas red (1:200) (Thermo Fisher, Waltham, MA, USA). After washing, the cells were permeabilized with 4% PFA (Sigma, St. Louis, MO, USA) for 10 min at RT followed by an incubation with primary antibodies for CB2 (1:300) (Abnova, Taipei, Taiwan) and p62 (1:300) (Sigma, St. Louis, MO, USA), for 20 min at RT in DPBS (PAN BIOTECH, Aidenbach, Germany). Next, secondary antibodies (1:2000) were incubated for 1 h at RT, and cells were then washed and mounted with DAPI (1:5000) (ThermoFisher, Waltham, MA, USA).

### 2.6. Imaging and 3D Reconstruction Modeling

Four colors confocal images were acquired from a confocal laser scan microscope (Leica TCS SP8). As excitation, four laser lines, 405, 488, 561, and 633 nm were primarily employed and, for the excitation and detection of the fluorescent signal, a 63xNA objective lens was used. The fluorescent signal was directed to a HyD after being spectrally separated. All the post-processing analysis and evaluation for images were performed using the software Imaris. A segmentation algorithm was used to isolate a structure of the CB2 co-localization with p62. Segmented images were than displayed and image snapshots were taken.

### 2.7. Fluorescence-Activated Cell Sorting (FACS) Analysis

The following antihuman antibodies were used to stain cell surface markers to establish the CIK phenotype: CD3-fluorescein isothiocyanate (FITC) (Biolegend, San Diego, CA, USA), CD56-phycoerythrin (PE) (Biolegend, San Diego, CA, USA), CD4-allophycocyanin (APC) (Biolegend), CD8-Brilliant Violet 421(BV421) (Biolegend, San Diego, CA, USA), CD3-phycoerythrin (PE) (Biolegend, San Diego, CA, USA), CD56-allophycocyanin (APC) (Biolegend, San Diego, CA, USA), and CD20-Pacific Blue (Biolegend, San Diego, CA, USA). For the surface and intracellular CB2 receptor staining, CIK cells and tumor cells were fixed and permeabilized with Invitrogen Intracelluar Fix & Perm set kit (ThermoFisher, Waltham, MA, USA), according to the manufacturer’s instructions. The cells were then stained with a FITC-conjugated antibody against CB2 (Cayman Chemical, City, MI, USA) and anti-rabbit IgG FITC-conjugated anti-CB2 antibody (Cayman Chemical, City, MI, USA). For intracellular p-38 and p62 proteins staining, cells were stained with a PE-conjugated antibody against p-38 MAPKs (ThermoFisher, Waltham, MA, USA) and AlexaFluor488-conjugated antibody against p62/SQSTM1 (JSR Life Sciences, Sunnyvale, CA, USA). 7-Aminoactinomycin D (7-AAD) (Biolegend, San Diego, CA, USA) was used to stain the dead tumor cells. To assess the cytotoxicity of CIK cells in combination with pure cannabidiol (Santa Cruz Biotechnologie, Heidelberg, Germany) in cell lines, the carboxyfluorescein succinimidyl ester (CFSE; ThermoFisher, Waltham, MA, USA)-labeled multiple pancreatic and multiple myeloma cancer cells were incubated along with Far Red (ThermoFisher, Waltham, MA, USA.)-labeled CIK cells in an E:T ratio of 10:1 and exposed to different concentrations (1–20 μM, 24 h at 37 °C) of pure cannabidiol (CBD). Pure cannabidiol was first solved in DMSO and afterwards diluted within the corresponding RPMI medium (PAN BIOTECH, Aidenbach, Germany). The cell suspensions were washed with DPBS (PAN BIOTECH, Aidenbach, Germany) twice. Finally, the dead cells were stained with Hoechst 33258 (Cayman Chemical, City, MI, USA) and Precision Count Beads (Biolegend, San Diego, CA, USA) were added.

### 2.8. CREB Phosphorylation Assay (p-CREB)

CIK cells at 14 days of culture were pelleted by centrifugation for 5 min at 1500 rpm, resuspended in cell culture medium and seeded at 1 × 10^7^ cells/mL (200 μL of cells per well) in 96-well tissue culture treated plates. Plates were incubated at 37 °C in a humidified atmosphere with 5% CO_2_ for 2 h and incubated 30 min prior to adding of pure cannabidiol (CBD, 100%) (Santa Cruz Biotechnologie, Heidelberg, Germany) from 1 μM to 20 μM. At the end of the incubation, cells were lysed by the addition of lysis buffer on a plate shaker for 10 min at RT. p-CREB detection was performed using the AlphaLISA SureFire Ultra p-CREB (ser133) assay kit (PerkinElmer, Waltham, MA, USA). The signal was detected on a SpectraMax plate reader with AlphaLISA-compatible filters.

## 3. Results

### 3.1. IL-2 Primarily Determines the Expression of CB2 Receptor on CIK Cells

**Figure 1:**
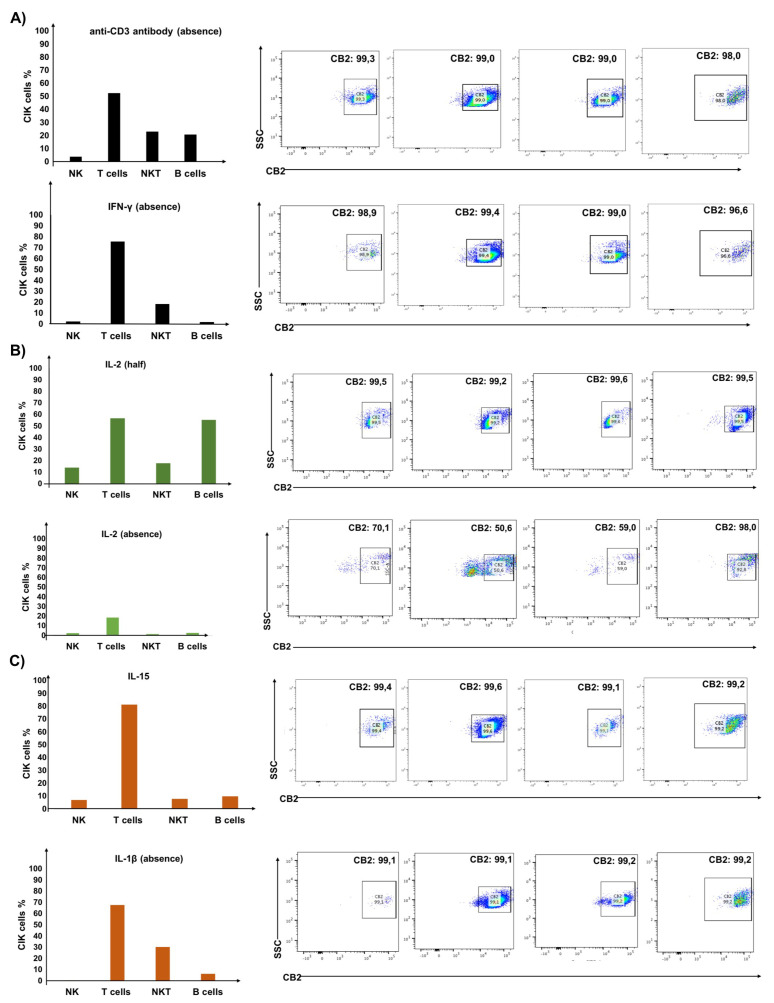
Differential expression of CB2 receptor in the cell subsets of CIK cells. CIK cells were cultured (**A**) in the absence of anti–CD3 antibody (**upper** panel) and IFN–γ (**lower** panel), (**B**) in half of the amount of IL–2 (**upper** panel) and in the absence of IL–2 (**lower** panel), and (**C**) with IL–15 (**upper** panel) and in the absence of IL–1β (**lower** panel). In all experiments, CIK cells were immunophenotyped at day 14 by flow cytometry and the differential expression of CB2 receptor in the main cell subsets of human CIK cells, i.e., NKT, T, NK, and B cells were determined.

CIK cells were generated from the PBMCs of healthy volunteers and phenotypes of CD3^+^CD56^+^ (NKT), CD3^+^CD56^−^ (T cells) and CD3^−^CD56^+^ (NK) were confirmed to see both surface and intracellular CB2 expression. Primarily, we observed CD3^+^CD56^+^ (23.1%), CD3^+^CD56^−^ (52.5%), and CD3^−^CD56^+^ (21.0%) CIK cells without anti-CD3 antibody and CD3^+^CD56^+^ (18.5%), CD3^+^CD56^−^ (75.6%), and CD3^−^CD56^+^ (2.58%) cells tested in the absence of IFN-γ (Figure 1). In addition, 3.92% CD20^+^ B cells without anti-CD3 antibody (Figure 1A) and 1.99% without IFN-γ were detected.

It is worth noting that the percentage of CB2-positive cells within these different subgroups did not differ significantly and remained completely positive. Of interest, when we used half of the amount of IL-2, the percentages of CD3^+^CD56^+^ (NKT), CD3^+^CD56^−^ (T cells) and CD3^−^CD56^+^ (NK) cells were 17.8%, 56.9%, and 14.0%, while in complete absence of IL-2, they were 30.4%, 67.7%, and 0.49%, respectively (Figure 1B). Importantly, the percentages of CB2 positive cells within CD3^+^CD56^+^ (59.0%), CD3^+^CD56^−^ (50.6%), and CD3^−^CD56^+^ (70.1%) decreased significantly in the complete absence of IL-2, while it remains entirely positive when only half of the amount of IL-2 was used. In addition, 55.3% CD20^+^ B cells were detected with half of the amount and 2.72% by a complete lack of IL-2; however, the percentages of CB2 positive cells remain high in both conditions. We extended our analysis by testing IL-15 or no IL-1β in a similar experimental setup and observed CD3^+^CD56^+^ (8.02%), CD3^+^CD56^−^ (81.3%), and CD3^−^CD56^+^ (7.07%) CIK cells with IL-15 and CD3^+^CD56^+^ (30.04%), CD3^+^CD56^−^ (67.7%), and CD3^−^CD56^+^ (0.49%) cells in the absence of IL-1β (Figure 1C). In this case, 10.0% CD20^+^ B cells were detected with IL-15 and 6.22% by a complete lack of IL-1β. Here again, the percentages of CB2 positive cells remain high in both conditions—thus indicating that IL-2 primarily determines the expression of the CB2 receptor on CIK cells.

### 3.2. An Autophagosomal-Associated Scaffold Protein p62 Also Colocalizes with CB2 Receptors in CIK Cells and the PANC-1 Cell Line

**Figure 2:**
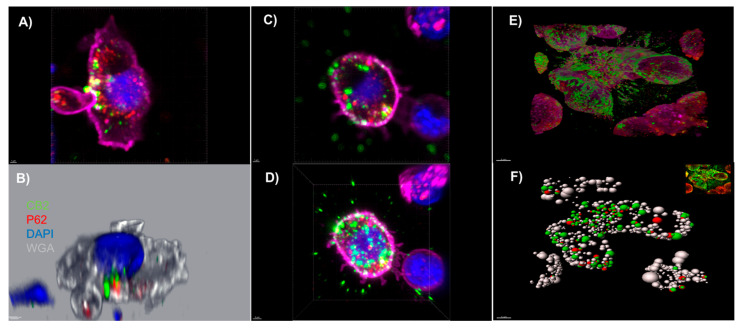
(**A**–**D**) Confocal light microscopy of CIK cells at day 14 expressing CB2 receptors. Immunohistochemical staining of CB2 (green), p62 (red), and DAPI (blue). The membrane was stained by Texas-red conjugated wheat-germ-agglutinin (magenta) and DAPI revealed the localization of the nucleus. (**A**,**B**) Co-localization of CB2 receptors and p62 vesicles at the cell membrane which are in yellow due to the overlap of green and red. 3D reconstruction of confocal images to distinguish the CB2 receptor (green) out of the membrane (grey), and p62 vesicles (red) inside the membrane in close proximity to CB2 receptors; (**C**,**D**) co-localization of CB2 receptors and p62 vesicles intracellularly. The CB2 receptors have a different distribution in the cell membrane; (**E**,**F**) confocal light microscopy of PANC-1 pancreatic cancer cell line expressing CB2 receptors. Immunohistochemical staining of CB2 (green), p62 (red). The membrane was stained by Texas-red conjugated wheat-germ-agglutinin (magenta). 3D reconstruction modeling of confocal images using a segmentation algorithm to distinguish a single signal from different channels showing co-localization of CB2 receptors and p62 vesicles.

As aforementioned, CB2 receptors colocalize with p62 vesicles mainly in the plasma membrane of transiently transfected HEK293 cells. Herein, we also investigated possible co-localizations of these two proteins in CIK cells and PANC-1 cells by using immunocytochemical studies and a 3D reconstruction modeling of confocal images. We observed that p62 vesicles were surrounded by CB2-positive areas (Figure 2). Particularly in CIK cells, reconstruction images showed that p62 vesicles were surrounded by CB2-positive areas at the surface membrane and also intracellularly (Figure 2A–D). In the case of PANC-1 cells, we used a segmentation algorithm and determined p62-positive regions that co-expressed CB2 (Figure 2E,F).

### 3.3. CIK Cells Showed a Low Level of Intracellular p-p38 and Donor Specific Variability in p-CREB

**Figure 3:**
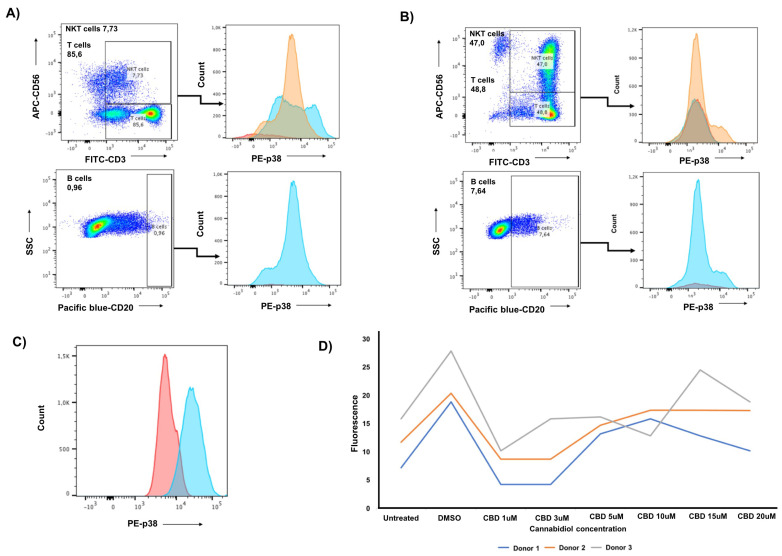
(**A**) The expression of p-p38 in the main cell subsets of human Cytokine-induced killer (CIK) cells, i.e., CD3^+^CD56^+^(NKT), CD3^+^CD56^−^ (T cells), and CD20^+^ B cells. Intracellular p–p38 expression was evaluated and the CIK cells were immunophenotyped at day 7 by flow cytometry. For the subsets CD3^+^CD56^+^(NKT) 7.73%, CD3^+^CD56^−^ (T cells) 85.6%, p–p38 expression (histogram representation) was 0% (red) and 3.0% (blue), respectively. The isotype is orange. For CD20^+^ B cells 0.96%, p–p38 expression was 0% (red). The isotype is blue. (**B**) Intracellular p–p38 expression was evaluated and the CIK cells were immunophenotyped at day 14 by flow cytometry. For the subsets CD3^+^CD56^+^(NKT) 47.0%, CD3^+^CD56^−^ (T cells) 48.8%, p–p38 expression (histogram representation) was % 0 (red) and 0% (blue), respectively. The isotype is orange. For CD20^+^ B cells 7,64%, p–p38 expression was 0% (red). The isotype is blue. (**C**) The expression of p–p38 in PANC-1 cell line. Intracellular p–p38 expression was evaluated, and the cells were immunophenotyped by flow cytometry. Histogram dotplot with PANC–1 cells (red) and isotype (blue). P–p38 was not detectable in PANC–1 cell line via flow cytometry. (**D**) Phosphorylation of CREB in CIK cells stimulated with cannabidiol from 1 μM to 20 μM for 30 min at 37 °C. The graph shows the mean of three independent experiments performed in technical triplicate, each with cells from a separate subject (three subjects in total). The data were normalized.

To determine potential effects of downstream mediators of CB2, such as phosphorylation of p38 and CREB, which presumably could indirectly affect the pleiotropic cytokines (IL-6/IL-10) that overlap with the CIK cell function. We next examined intracellular p-p38 expression in CIK cells immunophenotyped on days 7 and 14 of ex vivo expansion by flow cytometry (Figure 3). Interestingly, p-p38 was detectable only at low levels in the subset of T lymphocytes at day 7, whereas it was undetectable in other subsets at either day 7 or day 14 (Figure 3A,B). Similarly, we immunophenotyped PANC-1 cells and observed p-38 undetectable (Figure 3C). In the case of the second mediator (p-CREB), we incubated CIK cells (day 14) with cannabidiol at different concentrations of 1–20 μM and found a weak signal (Figure 3D). Notably, in the case of p-CREB, donor-specific variability was observed, with two donors showing a decrease in CREB phosphorylation at 1–3 μM and one at 1 μM compared to the untreated and DMSO control.

### 3.4. Cannabidiol Significantly Decreases the Viability of PANC-1 Cells and Significantly Increases the Cytotoxicity of CIK Cells against PANC-1 at Low Concentrations

**Figure 4:**
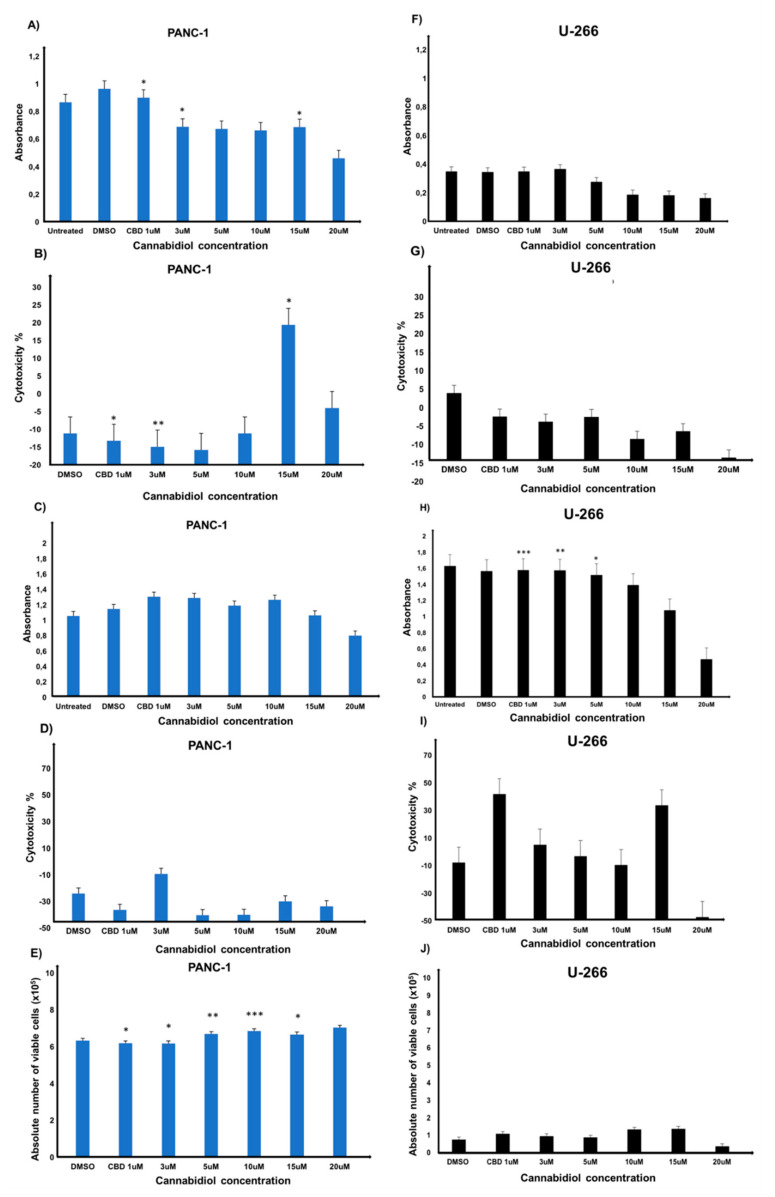
The cell viability in PANC–1 cells exposed to various concentrations of CBD by (**A**) CCK–8 assay and (**B**) LDH assay. The cell viability of the co–cultured CIKs and PANC–1 cells at E:T ratio of 10:1 by (**C**) CCK–8 assay and (**D**) LDH assay. (**E**) The absolute number of viable PANC–1 cells for each sample condition with cocultured CIKs and PANC–1 cells at an E:T ratio of 10:1. The cell viability in U–266 cells exposed to various concentrations of CBD by (**F**) CCK–8 assay and (**G**) LDH assay. The cell viability of the co–cultured CIKs and U–266 cells at E:T ratio of 10:1 by (**H**) CCK–8 assay and (**I**) LDH assay. (**J**) The absolute number of viable U–266 cells for each sample condition with cocultured CIKs and PANC–1 cells at an E:T ratio of 10:1 (* *p* < 0.05, ** *p* < 0.01, *** *p* < 0.001).

Next, we measured the cell viability of PANC-1 cells exposed to various concentrations of CBD (1–20 μM) for 24 h at 37 °C. We observed significant decrease in the cell viability compared to the DMSO control (Figure 4A). Of interest, when cytotoxicity of PANC-1 cells exposed to different concentrations of CBD was assessed, a significant decrease in LDH release was observed at low concentrations (1 μM and 3 μM) and an increase was observed at high concentrations (15 μM) compared to the DMSO control (Figure 4B). Notably, no significant difference was observed when effector cells were cultured with target cells (PANC-1) E:T ratio of 10:1 and exposed to different CBD concentrations (1–20 μM) (Figure 4C). The LDH cytotoxicity experiment also showed no significant differences compared to the DMSO control (Figure 4D).

However, when effector cells were cocultured with target cells (PANC-1) E:T ratio of 10:1 and exposed to different concentrations of CBD (1–20 μM) for 24 h at 37 °C, a concentration-dependent inhibitory response was observed using flow cytometry analysis (Figure 4E)—thus suggesting that CBD significantly decreases the viability of PANC-1 cells presumably by increasing the cytotoxicity of CIK cells.

To determine whether this low effective CBD dose, which appears to be sufficient to stimulate cytotoxic function of CIK cells, is limited to pancreatic cells or if it could be a general phenomenon in cancer, we additionally tested myeloma cells. Particularly, we measured the cell viability (Figure 4F) and cytotoxicity (Figure 4G) of U-266 cells exposed to various concentrations of CBD (1–20 μM) for 24 h at 37 °C. No significant difference was observed compared to the DMSO control in both experiments. However, a significant difference was observed when effector cells were cultured with target cells (U-266) E:T ratio of 10:1 and exposed to different CBD concentrations (1−20 μM) (Figure 4H). It is worth noting a significant increase at 1 μM and 3 μM and a significant decrease at 5 μM compared to the DMSO control was observed. However, it remains difficult to distinguish between tumor cells and CIK cells. Notably, no significant difference was observed in the LDH release (Figure 4I) when effector cells were cultured with target cells (U-266) E:T ratio of 10:1 and exposed to different CBD concentrations (1–20 μM) and using flow cytometry analysis (Figure 4J) compared to the DMSO control.

## 4. Discussion

It has been well understood that genetic-epigenetic, inter/intra-individual heterogeneity and various yet to be known factors contribute to the complexity of cancer [19,20,21]. Nevertheless, the relative contribution of several immunotherapeutic approaches has helped to partially tackle this adverse effect of disease in the clinics. Among these approaches, cytokine-induced killer (CIK) cell therapy has played a pivotal role and raised the bar regarding treatment response due to its safe and efficient methodology [22,23,24]. The uniqueness of CIK cells is their encouraging synergetic effect with cancer associated inhibitors/compounds in preclinical models since cannabinoids have been the subject of intensive cancer research, in particular cannabinoid receptor 2 (CB2) due to its expression in cells of the immune system where CIK cells also play an important role. To date, no study has thoroughly investigated the combinatorial impact of CBD and CIK cells, particularly in pancreatic cancer (PC). It is worth noting that the implication of cannabinoids in PC can be from different CB ligands from the study where the combination of synthetic cannabinoids and gemcitabine synergistically trigger the inhibition of PC cells growth by a ROS-mediated autophagy induction involving the AMP-activated protein kinase (AMPK) [25,26]. Considering this, herein, we sought to investigate whether inducing CIK cells with CBD can enhance their cytotoxicity in pancreatic cells. Besides our major focus on the PC cellular model (PANC-1 cell line), we also used myeloma cells (U-266 cell line) as a proof of concept.

In our analysis, we first found that IL-2 primarily determines the expression of CB2 receptor on CIK cells. This was clearly evident from the analyses when both surface and intracellular CB2 expression were confirmed on CIK cells generated from PBMCs of healthy volunteers and respective percentage of CD3^+^CD56^+^, CD3^+^CD56^−^, and CD3^−^CD56^+^ CIK cells was quite distinguishable in all groups. Importantly, the percentages of CB2 positive cells always remains high, regardless of co-culturing with any CIK cell mediators (anti-CD3 antibody, IFN-γ, IL-2, IL-15, and IL-1β). The cytokine cocktail includes IL-2 to promote survival and activation of cytolytic effector function of CIK cells, and IL-15, which is capable of further activating CIK cells and shares common signaling components with IL-2, e.g., activation of the Jak/STAT signaling pathways. Next, we investigated whether CB2 receptors colocalize with p62 vesicles in CIK cells and PANC-1 cells, as it has been previously reported in HEK293 cells [17]. Our analysis clearly showed that p62 vesicles were surrounded by CB2-positive areas at the surface membrane and also intracellularly in CIK cells, while p62-positive regions were co-expressed with CB2 in PANC-1 cells. Given that CIK cells are heterogenous, here it is unclear which subtype of CIK cells contributes predominantly towards the localization with p62. Previously, it has been shown that CB2 induces the phosphorylation of p38 MAPKs, downstream CREB phosphorylation and induction of IL-6, IL-10 cytokine secretion in human primary leukocytes [18]. Therefore, we also examined intracellular p-p38 expression in CIK cells immunophenotyped on day 7 and day 14 of ex vivo expansion. Interestingly, we found that CIK cells showed a low level of intracellular p-p38, primarily in the subset of T lymphocytes at day 7, while, in the case of p-CREB, we found a weak and variable signal among donors when CIK cells (day 14) were incubated with CBD. This clearly indicated that CBDs (mainly at low concentration) are sufficient to stimulate the cytotoxic function of CIK cells without exerting the downstream mediators like p38 and/or CREB, particularly in the pancreatic adenocarcinoma cell line. Nevertheless, whether the expression of other molecules such as perforin or granzyme B is also affected in this crosstalk between CIK, and CBD requires further validation. It is worth noting that cell viability and cytotoxicity of PANC-1 cells were also found to be significantly decreased when they were exposed to various concentrations of CBD (1–20 μM) for 24 h at 37 °C. Interestingly, when CIK cells were co-cultured, a concentration-dependent inhibitory response was observed in these cells—thus suggesting that CBD (in low concentration) presumably increased the cytotoxicity of CIK cells, which in turn negatively impacted the viability of PANC-1 cells. Despite this low effective CBD dose, impact is limited to pancreatic cells or it is a general phenomenon in cancer, we additionally tested myeloma cells. Of interest, like PC cells, a significant difference was observed when effector cells were cultured with target cells (U-266) E:T ratio of 10:1 and exposed to different CBD concentrations (1–20 μM)—hence confirming that a low dose of CBD impacted in a similar way in both PC and myeloma cells, presumably via CIK cells.

Here, it is also important to mention the limitation of the study like using multiple cell lines with a varied genetic background would provide more detailed insights. Certainly, an in vivo validation of CBD-CIK crosstalk in a preclinical model is warranted. Still, ours is the first study to show that a low dose of pure cannabidiol is sufficient to stimulate the cytotoxic function of CIK without exerting any associated mediator. As CIK cell therapy is safe, introducing pure cannabidiol particularly for the non-respondent patients may help to increase the therapeutic response.

## 5. Conclusions

A low dose of pure cannabidiol (CBD) is sufficient to stimulate the cytotoxic function of CIK cells in cancer cells, primarily in pancreatic and myeloma cells.

## Data Availability

Not applicable.
